# The Potential Effect of Insulin on AChE and Its Interactions with Rivastigmine In Vitro

**DOI:** 10.3390/ph14111136

**Published:** 2021-11-09

**Authors:** Tahereh Jamshidnejad-Tosaramandani, Soheila Kashanian, Mahsa Babaei, Mohamed H. Al-Sabri, Helgi B. Schiöth

**Affiliations:** 1 Nanobiotechnology Department, Faculty of Innovative Science and Technology, Razi University, Kermanshah 6714414971, Iran; T.jamshidnejad89@gmail.com; 2 Department of Biology, Faculty of Science, Razi University, Kermanshah 6714414971, Iran; babaeimahsa28@gmail.com; 3 Department of Neuroscience, Functional Pharmacology, University of Uppsala, BMC, Husargatan 3, Box 593, 751 24 Uppsala, Sweden; mohamed.alsabri@neuro.uu.se (M.H.A.-S.); Helgi.Schioth@neuro.uu.se (H.B.S.); 4 Nano Drug Delivery Research Center, Health Technology Institute, Kermanshah University of Medical Science, Kermanshah 6734667149, Iran; 5 Faculty of Chemistry, Sensor and Biosensor Research Center (SBRC), Razi University, Kermanshah 6714414971, Iran; 6 Institute for Translational Medicine and Biotechnology, I.M. Sechenov First Moscow State Medical University, Trubetskay Str. 8, bldg 2, 119991 Moscow, Russia

**Keywords:** Alzheimer disease, insulin, rivastigmine, acetylcholinesterase inhibition, combinational therapy

## Abstract

There is no definite cure for Alzheimer’s disease (AD) due to its multifactorial origin. Drugs that inhibit acetylcholinesterase (AChE), such as rivastigmine, are promising symptomatic treatments for AD. Emerging evidence suggests that insulin therapy can hinder several aspects of AD pathology. Insulin has been shown to modify the activity of AChE, but it is still unknown how insulin and AChE interact. Combination therapy, which targets several features of the disease based on existing medications, can provide a worthy therapy option for AD management. However, to date, no studies have examined the potential interaction of insulin with AChE and/or rivastigmine in vitro. In the present study, we employed the Response Surface Methodology (RSM) as an in vitro assessment to investigate the effect of insulin on both AChE activity and rivastigmine inhibitory action using a common spectrophotometric assay for cholinesterase activity, Ellman’s method. Our results showed that insulin, even at high concentrations, has an insignificant effect on both the activity of AChE and rivastigmine’s inhibitory action. The variance of our data is near zero, which means that the dispersion is negligible. However, to improve our understanding of the possible interaction of insulin and rivastigmine, or its target AChE, more in silico modelling and in vivo studies are needed.

## 1. Introduction

AD is a progressive and irreversible neurodegenerative disease with around 50 million cases worldwide, and 10 million new cases every year [1]. To date, there is no well-defined therapy option for AD [2]. AD is rapidly becoming a pressing public health and economic concern, with the global number of patients predicted to be more than 130 million by 2050 [3]. The cause of the common form of AD is still unknown, but a few forms of the disease are heritable, caused by mutations in specific genes [4]. It has been shown that the accumulation of extracellular insoluble β amyloid peptides (Aβ), intracellular hyper-phosphorylated tau protein neurofibrillary tangles (NFTs), the progressive degeneration of the cholinergic neurons, oxidative stress, and metabolic malfunctions in neural cells are among the most important cellular mechanisms in AD [5,6,7,8,9,10,11,12]. AD is usually characterized by a decline in cognitive functions that affects patients’ daily activities [13]. AD is fatal, and just a few symptomatic treatments are available due to the obscure origin of the disease [14,15].

Due to its multifactorial origin, current therapeutic approaches have failed to address the root cause of AD and are therefore employed for symptomatic treatment [16,17,18]. The only available therapeutics for AD in the market works on the cholinergic pathway or the N-Methyl-D-Aspartate (NMDA) receptor [19]. However, combination therapy using the existing medications to form multifunctional agents as advanced drug delivery systems or prodrugs that can target several features of the disease can provide huge progress in AD treatment [20,21,22,23,24]. For example, combinations of metal chelation, neuroprotective antioxidants, Aβ anti-aggregations, cholinergic modification, and anti-inflammatory agents into a single drug delivery system yield significant enhancement in AD management [25,26,27,28,29,30].

**Figure 1:**
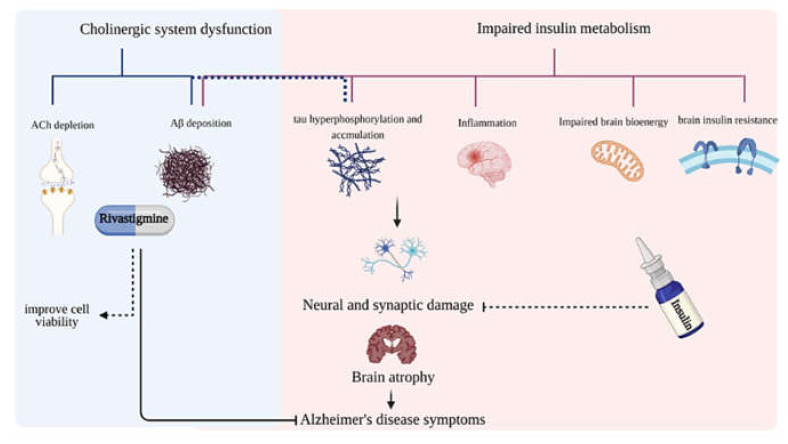
A summary of the roles of rivastigmine and insulin in AD treatment based on cholinergic hypothesis and distorted cerebral insulin metabolism. The onset and progression of AD symptoms are in close relation to some cellular and molecular hallmarks: the accumulation of extracellular insoluble Aβ, intracellular hyper-phosphorylated NFTs, inflammation, insulin resistance, and metabolic malfunction in brain cells, and the progressive degeneration of the cholinergic neurons. AChE can also accelerate Aβ deposition and the accumulation of senile plaques. The loss of function in neurons and synapses is responsible for the incidence of AD symptoms. Combination therapy with rivastigmine and insulin can target various features of AD. Rivastigmine blocks ACh hydrolysis, elevates synaptic ACh levels, suppresses Aβ deposition, and promotes cholinergic function. Insulin enhances brain bioenergy, synaptogenesis, and synaptic remodelling, improves dendritic spine formation, boosts turnover of neurotransmitters, influences the clearance of Aβ and the phosphorylation of tau.

Designing combination therapy to target multiple aspects of AD pathology is essential since so far, the therapeutic paradigm of “one compound, one target” has failed [31]. Rivastigmine is both a pseudo-irreversible acetylcholinesterase inhibitor (AChEI) and a butyrylcholinesterase inhibitor (BuChE I), effective in the symptomatic treatment of mild to moderate dementia in AD and Parkinson’s disease (PD) [32,33,34]. AChEIs block Acetylcholine (ACh) hydrolysis, elevate synaptic ACh levels, promote cholinergic function, and inhibit the secondary effect of AChE activity on Aβ deposition [35,36,37]. Moreover, investigations on other AD treatment options are still in progress [38]. Studies over time have shown that enhanced CNS insulin can provide therapeutic benefits to AD patients [39,40,41,42,43]; however, its exact effect on cognitive functions is rather unclear [44]. Insulin enhances glucose metabolism in the brain, improves synaptogenesis and neurotransmitter turnover, and ameliorates Aβ clearance and phosphorylation of tau [44,45]. Therefore, rivastigmine along with insulin could comprise a promising combined therapy for AD (Figure 1). Further studies are needed to investigate the interacting effects of insulin with AChEI as repurposed combination therapy for AD management.

Studies have reported that insulin can affect the enzyme activity of AChE, but the results are inconclusive. For example, a study by Agrawal et al. showed an anticholinesterase effect of insulin and melatonin in the brain of amnesic mice [46]. Additionally, a more recent study reported that insulin treatment is associated with a reduction of AChE activity in the hippocampus and the frontal cortex regions of the rat brains [47]. S. Lakhman showed a significant rise in AChE activity from the isolated regions of diabetic rat brains. The same study demonstrated that acute hyperglycemia elevates AChE activity, and insulin administration reversed this effect [48]. On the other hand, several studies have reported that insulin can enhance AChE activity [49,50,51].

Insulin, which is a peptide hormone, could interact directly with AChE since in vitro studies have reported non-specific interactions of different peptides with AChE or its inhibitors [52,53,54]. For example, Shamsi et al. has reported an interaction between rivastigmine and human transferrin, a protein with a molecular weight of around 80 kDa, through a formation of a drug-protein static complex [54]. In other in vitro studies, different kinds of proteins such as a cysteine protease glycoprotein (ZCPG), aflatoxin B (AFB), anchovy protein hydrolysate (APH), and fasciculin 2 have been reported to interact and inhibit AChE [55,56,57,58]. Interestingly, hemp seed protein hydrolysates can inhibit AChE activity, and the amino acid composition of the hydrolysates remarkably affects the enzyme’s inhibitory potential due to its alternative interactions with the peripheral anionic site (PAS) of the AChE [59]. Additionally, Z. Yu et al. showed both in vitro and in silico studies that the *Salmo salar* roe protein-derived peptide, named WIR, exhibited potent inhibition against AChE. The same study indicated that WIR can bind to both PAS and the catalytic anionic site (CAS) of AChE by hydrogen bond and pi–alkyl interactions [60]. Similarly, insulin, which is a charged protein with a molecular weight of 5.81 kDa, might have such interactions with AChE or its inhibitors such as rivastigmine. 

Overall, elucidating protein-drug interactions between insulin and rivastigmine or its target AChE is essential in the pharmacological profiling of repurposed combination therapy. To date, no in silico nor in vitro studies have examined the possible interactions of insulin with rivastigmine, or with its target AChE. Hence, the current study aimed to conduct in vitro assessments of the effect of insulin on AChE activity or rivastigmine’s inhibitory action.

## 2. Results

### 2.1. Determination of Kinetics for ATCh and Rivastigmine to Study Insulin Effects on AChE Activity

To measure the effect of insulin on AChE activity and rivastigmine’s inhibitory action, we first inquired into the optimized substrate (ATCh) and inhibitor (rivastigmine) concentrations needed to design basic enzymatic tests in the absence of insulin. We started by measuring AChE activity and rivastigmine inhibitory action through a spectrophotometric analysis based on Ellman’s method [61], and calculated all the parameters based on Michaelis–Menten kinetics. Initially, substrate (ATCh) concentration was increased to determine the concentration at which AChE was the most active. In the case of AChE, maximum enzyme activity was reached between 100 µM and 1 mM of ATCh (Figure S1). Accordingly, the determined 500 µM concentration of ATCh was chosen for the determination of rivastigmine IC50. Our result showed that an increase in rivastigmine concentrations caused a reduction in enzymatic reaction rate up to 1200 µM, above which there was no change (Figure S2). The rivastigmine inhibition type for AChE was pseudo-irreversible.

Next, we asked whether different concentrations of insulin could affect AChE activity. The 0–50 µM concentrations of insulin were added to the basic enzymatic reaction with the determined ATCh concentration (500 µM), which was subsequently evaluated according to Ellman’s method [61] and Michaelis–Menten kinetics. No significant effect was observed, even at high concentrations of insulin. As can be seen, the variance of our data is near zero (0.00001), which means that the dispersion of our data is negligible and therefore the effect of insulin on the enzyme activity is insignificant (Figure S3). Since the enzyme function is highly related to an active site and substrate structures, insulin alone most probably cannot easily change the structure-function relationship, which explains our result. Thus, the modulatory effect of insulin on the cholinergic system indicated in the literature might be explained by insulin-induced hypoglycemia in different brain regions, which causes a significant decrease in AChE activity [62], and by the insulin-enhancing effect on ACh synthesis through stimulating the expression of choline acetyltransferase [63]. Since the direct influence of insulin on AChE activity is unlikely based on our result, the precise mechanisms of insulin interaction with AChE remain to be determined by further studies.

### 2.2. RSM Studies on the Effect of Different ATCh, Rivastigmine, and Insulin Concentrations on AChE Activity

An RSM was carried out to obtain more information about the integrated effects of different rivastigmine and insulin concentrations on AChE activity. To examine the effect of insulin on AChE activity and rivastigmine inhibitory action under a close-to-real synaptic condition, different ATCh concentrations were considered in the study design. All the tests were conducted via spectrophotometer according to Ellman’s method [61].

#### 2.2.1. Integrated Effect of Rivastigmine and ATCh

**Figure 2:**
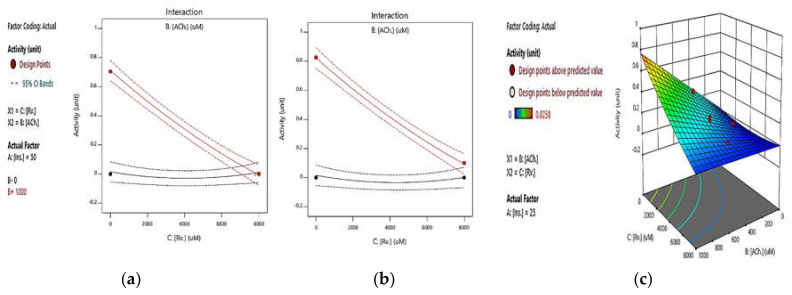
RSM plots of the integrated effects of rivastigmine and ATCh (**a**) at concentrations of 0 and (**b**) 50 µM of insulin (in both diagrams A and B, the red and black curves represent ATCh concentrations of 1000 and 0 µM, respectively). (**c**) A 3D response surface plot illustrating the integrated effect of rivastigmine and ATCh at an insulin concentration of 25 µM, all based on the standard Ellman’s method (*p*-value > 0.0001).

First, in order to investigate the integrated effect of rivastigmine and ATCh, with a constant concentration of insulin, we conducted enzymatic reaction tests at insulin concentrations of 0 and 50 µM based on Ellman’s method (Figure 2a,b). In both diagrams, the red and black curves are related to the ATCh concentrations of 1000 and 0 µM, respectively. As expected, enzyme activity was notably reduced by the increase in the concentration of rivastigmine, an effective AChEI. As in Figure 2a,b, there was no AChE activity in the absence of ATCh, the substrate. However, in both figures and for the ATCh concentration of 1000 µM (red curves), the rivastigmine inhibitory effect was sustained in the presence or absence of insulin, at all measuring points, indicating that neither AChE activity nor the rivastigmine inhibitory effect were influenced by the presence of insulin. According to the *p*-value (>0.0001), these responses are significant. Figure 2c also shows the integrated effect of rivastigmine and ATCh at an insulin concentration of 25 µM in a 3D plot.

#### 2.2.2. Integrated Effect of Insulin and ATCh

**Figure 3:**
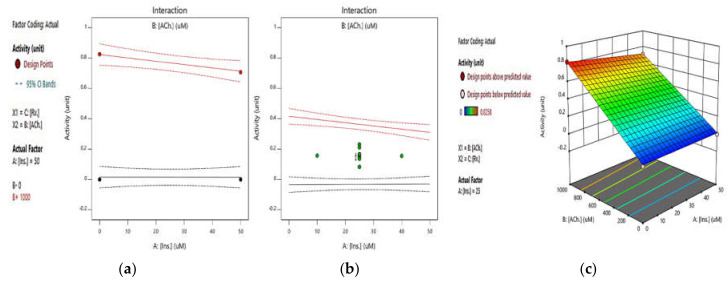
RSM plots for the integrated effect of insulin and ATCh at concentrations of (**a**) 0 and (**b**) 4000 µM of rivastigmine (in both diagrams A and B, the red and black curves represent ATCh concentrations of 1000 and 0 µM, respectively). (**c**) A 3D response surface plot illustrates the integrated effect of insulin and ATCh at a rivastigmine concentration of 0 µM, whereby insulin has an insignificant effect on AChE activity. All the tests were conducted based on the standard Ellman’s method (*p*-value = 0.039).

Second, the integrated effect of insulin and ATCh, with a constant concentration of rivastigmine, was evaluated by the standard Ellman’s method. Figure 3a,b shows the integrated effect of insulin and ATCh at concentrations of 0 and 4000 µM of rivastigmine, respectively. In both diagrams, the red and black curves are related to ATCh concentrations of 1000 and 0 µM, respectively. In Figure 3a,b, in the absence of the substrate (black curves), there was no AChE activity. Nevertheless, at the concentration of 1000 µM for ATCh (red curves), AChE activity decreased only by 2% and 3% in the absence and presence of rivastigmine, respectively, indicating that insulin, even at the highest concentration (50 µM), could not noticeably alter AChE activity. According to the ANOVA Table, the *p*-value of this result was 0.039 and statistically significant, thus confirming our results which showed a negligible effect of insulin on AChE activity (Figure S3). Figure 3c also shows the integrated effect of insulin and ATCh in the absence of rivastigmine (0 µM).

#### 2.2.3. Integrated Effect of Insulin and Rivastigmine

**Figure 4:**
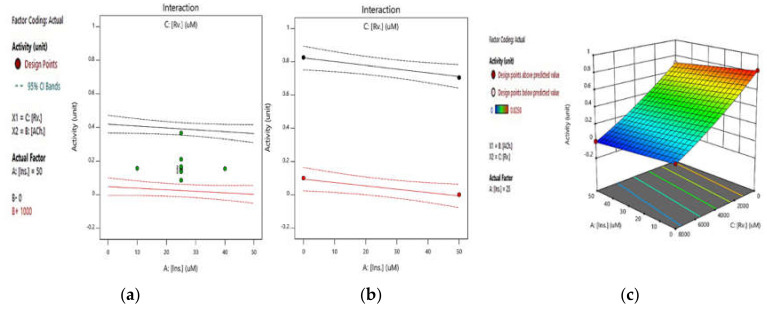
RSM plots of the integrated effect of insulin and rivastigmine at concentrations of (**a**) 500 and (**b**) 1000 µM of ATCh (in both diagrams, the red and black curves represent rivastigmine concentrations of 8000 and 0 µM, respectively). (**c**) A 3D response surface plot illustrates the integrated effect of insulin and rivastigmine at 1000 µM of ATCh concentration, whereby the increase in insulin concentration has an insignificant effect on the enzyme activity. All the tests were conducted based on the standard Ellman’s method (*p*-value = 0.8346).

Next, the integrated effect of insulin and rivastigmine at a constant concentration of ATCh was measured. Figure 4a,b shows the integrated effects of insulin and rivastigmine at concentrations of 500 and 1000 µM of ATCh, respectively. In both diagrams, the red and black curves are for rivastigmine concentrations of 8000 and 0 µM, respectively. As in Figure 4a, with a constant concentration of the substrate in the presence and absence of rivastigmine, the decreasing effect of insulin on AChE activity was only 2%, and the presence of rivastigmine did not alter this effect. Additionally, in Figure 4b, in the presence and absence of the rivastigmine, there was only a 3%reduction in enzyme activity. According to the ANOVA Table, the *p*-value = 0.8346 indicates that the interaction between rivastigmine and insulin is not significant. Figure 4c also shows the integrated effect of insulin and rivastigmine at ATCh concentrations of 1000 µM in a 3D plot.

## 3. Discussion

Both brain insulin metabolism and AChE activity are good targets for AD treatment [37,64]. AChEIs have been widely studied in the context of their efficacy in the treatment of AD through their inhibitory action on AChE; however, their interaction effects with insulin have not yet been elucidated. This is very important since insulin plays a crucial role in synaptic plasticity and memory function as well as in AD onset and progression [65], and studies have shown that insulin can alter AChE activity, the target of AChEIs. Moreover, whether insulin can enhance or diminish AChE activity is still under debate, and the suggested underlying mechanisms are speculative or inconclusive [46,47,48]. Interestingly, in addition to their promising effect on AD treatment, it has been shown that AChEIs can enhance insulin secretion [66]. Given this and the possible synergetic effects of insulin and AChEIs in AD management, it is essential to study the drug-protein interaction between insulin and AChEIs and their target, AChE, before repurposing them as a combination therapy for AD. 

The present study elucidates whether the insulin effects on AChE activity suggested in the literature were initiated by the direct interaction of insulin with AChE. Our findings showed that insulin has no significant effect on AChE activity in vitro. A direct evaluation of the insulin effect on AChE activity indicates that ascending concentrations of insulin have a negligible effect on AChE activity (See Figure S3), which was confirmed by the RSM results (See Figure 2a,b and Figure 3c). Additionally, the RSM results indicated that insulin has a negligible suppressing effect on AChE activity in the presence or absence of rivastigmine (See Figure 3a,b). Hence, the insulin-modulating effect on AChE activity mentioned in the literature might be initiated through other indirect mechanisms.

One possibility is that insulin might affect AChE activity through the regulation of RNA levels as insulin has been shown to alter the microRNA profiles of different proteins involved in AD pathogenesis in cell cultures and animal models [67]. For example, Fishwick et al. showed that the acute exposure of depolarized neuronal cells to insulin temporarily triggers increased levels of high-affinity choline transporter proteins, which is attenuated by chronic insulin exposure [68]. It could be speculated that insulin might decrease AChE expression in the brain, which could be further affected under the co-administration of insulin and rivastigmine. Thus, it would be interesting to investigate such a mechanism in vivo using neural cultured cells or mouse models. 

The study also demonstrates that there seem to be no direct drug interactions between insulin and rivastigmine’s inhibitory effect on AChE, since the inhibitory activity of rivastigmine on AChE did not change even at a high concentration of insulin (See Figure 4a,b). Accordingly, these results suggest no drug-protein interaction between insulin and rivastigmine in vitro. This might provide crucial information for the pharmacological profiling of both drugs while designing a combination therapy. Nevertheless, further studies that include in silico modelling are recommended in order to develop these findings.

Our findings should be interpreted in the context of limitations. In vitro enzymatic tests on electric eel AChE have the rather simplistic view of not considering other components, metabolites, and factors that are present in the synaptic cleft as those under in vivo conditions in different species [69,70]. However, we conducted a logical RSM to study the effect of insulin considering all of the main factors involved in the cholinergic system.

## 4. Materials and Methods

### 4.1. Materials 

Acetylcholinesterase, lyophilized from electric eel, Acetylthiocholine (ATCh) iodide, and 5,5 -dithiobis-2- nitrobenzoic acid, DTNB Ellman’s reagent, were all purchased from Sigma, CA, USA. Rivastigmine tartrate was purchased from Daroupakhsh Co. (Tehran, Iran). Human insulin was kindly provided by RONAK DAROO Company (Markazi, Iran) as a gift.

### 4.2. Methods

#### 4.2.1. Determination of Kinetics for ATCh and Rivastigmine to Study Insulin Effect on AChE Activity

To find the kinetic model for AChE inhibition, the enzymatic reaction rate of AChE was determined by Ellman’s method [61]. It is based on the fact that thiocholine reacts immediately, quantitatively, and irreversibly to Ellman’s reagent, 5,5′-dithiobis-(2-nitrobenzoic acid) (DTNB), forming a yellow product (5-mercapto-2-nitrobenzoic acid). We measured the absorbance (*A*) versus time (*t*) at pH 7.4 in phosphate-buffered saline (PBS) and at room temperature, with a constant wavelength of 412 nm using a UV-VIS spectrophotometer (Carry, Australia). The kinetic studies were conducted using a quartz cuvette as a reactor. The cuvette was filled with chosen volumes of PBS, a determined excess amount of DTNB, ascending volumes of substrate solutions (ATCh) for K_m_ evaluation, and ascending volumes of the inhibitor (rivastigmine) for a half-maximal inhibitory concentration (IC50), which was determined based on Michaelis–Menten kinetics. The reaction was started by the fast (<1 s) pipetting of the chosen volume for the AChE solution with the vigorously mixed reaction mixture. The values of *A* were continuously measured vs. *t* over 1 min, and subsequently saved in a lab PC. Then, we evaluate the influence of the different concentrations of insulin (0–50 μM) on AChE activity with the same method for investigating the possible alternation of AChE activity.

#### 4.2.2. RSM Studies on the Effect of Different Atch, Rivastigmine, and Insulin Concentrations on AChE Activity

RSM studies [71] on different ATCh, rivastigmine, and insulin concentrations were conducted to simulate the comprehensive effect of insulin under close-to-real conditions in a synaptic cleft. The effect of three quantitative variables was evaluated based on RSM by Central Composite Design (CCD) using the design expert (DoE) software 10 State Ease (Minneapolis, MN). AChE activity was the corresponding response of three variables: A = (insulin), B = (ATCh), and C = (rivastigmine). For each factor, five different levels and the following points were selected for 20 runs of the experiments, using CCD (Table S1).

The quadratic model was chosen as the best model to define the factor responses (Table S2). Analysis of variance (ANOVA) was used to evaluate the significance of the quadratic regression model. Moreover, the model terms were assessed using the *p*-value with a 95% confidence level. The coefficient parameters were assessed by response surface regression analysis using the software DoE. Additionally, it was applied to obtain the residuals, three-dimensional (3D) surface, and two-dimensional (2D) contour plots of the response models (Table S3).

From Table S3, the quadratic model developed from the RSM was statistically significant for AChE activity (Y). The low value of P > F (less than 0.05) indicated the randomness of the results and the significant effect of the model terms on the response. The “Lack of Fit *p*-value” of 0.2 implied that the Lack of Fit was not significant. The quadratic model was used to explain the mathematical relationship between the independent variables and the dependent response. The mathematical expression for the relationship between AChE inhibition, as a corresponding response, and the three variables of insulin (A), ATCh (B), and rivastigmine (C) should be calculated according to the following Equation (1):

(1) $$R=A+B+C+\mathrm{AB}+\mathrm{AC}+\mathrm{BC}+A^{2}+B^{2}+C^{2}$$

Statistical analysis showed that the coefficients AC, A^2^, and B^2^ were statistically not significant. Therefore, A^2^ and B^2^ were omitted from the model. However, since the study purpose was to evaluate the simultaneous effect of insulin (variable A) and rivastigmine (variable C) on the enzyme activity, AC was not omitted from the model, although it had the *p*-value > 0.1 (Table S2). The final model was obtained in both coded and non-coded forms (Tables S4 and S5). The F value shown in Table S2 indicates that ATCh (F value = 311.88) had the greatest effect on the enzyme activity, followed by rivastigmine and insulin. It can be explained by the fact that ATCh is the enzyme substrate; thus, the enzyme activity is highly dependent on this factor. Furthermore, the interaction between ATCh and rivastigmine showed a high F value of 235.23. Following the same logic, since rivastigmine is an effective AChE inhibitor, the effect of this factor was expected to be considerable. The insulin F-value was 5.59 and the *p*-value was 0.039, which was less than 0.05 and thus still considered significant. The new equation was modified to:

(2) $$R=R_{0}+A+B+C+\mathrm{AB}+\mathrm{AC}+\mathrm{BC}+C^{2}$$

The high value of the coefficient of determination (R^2^ = 0.9838) is a measure of the goodness-of-fit to the model. This indicates a high degree of correlation between the predicted response and the experimental responses. The adjusted R^2^ = 0.9743 also confirmed the high correlation between the observed and theoretical values. From Figure S4, it was also confirmed that the experimental (actual) values were very close to the predicted ones.

The Adeq Precision value indicates the signal-to-noise ratio, and the optimal value is greater than 4. As can be seen, the Adeq precision value for the proposed model was 37.15, which indicates an excellent signal-to-noise ratio, signifying that the proposed model can be used to predict points in the design-covered area. At last, the ANOVA of each response was evaluated, and the effects of the independent variables were expressed in 3D response plots.

## 5. Conclusions

Our study demonstrates that insulin has no direct effect on either AChE activity or the rivastigmine inhibitory action in vitro. This may rule out possible direct interactions between insulin and AChE, suggesting other indirect mechanisms for the cross-talk between insulin and AChE. Most importantly, given the potential benefits of insulin in the management of AD, concurrent use of insulin and rivastigmine as a single drug delivery system, perhaps as a prodrug or as an adjunct treatment, could be considered since the inhibitory action of rivastigmine on AChE is preserved even at considerable concentrations of insulin in vitro. Nevertheless, more in vivo studies using neural cells or sophisticated animal models are needed to elucidate possible drug interactions and potential synergetic effects of insulin and rivastigmine as a combination therapy for AD management.

## Data Availability

Data is contained in the article and Supplementary Materials.

## Supplementary Materials

The following are available online at https://www.mdpi.com/article/10.3390/ph14111136/s1, Table S1: CCD matrix for the inhibition of AChE activity as the corresponding response of three variables (A = (insulin), B = (ATCh), and C = (rivastigmine)), Table S2: ANOVA data of the quadratic model, Table S3: Quadratic model as the best model to define the factor responses, Table S4: Coded form of the final model, Table S5. Non-coded form of the final model (Actual), Figure S1: Km determination for ATCh, Figure S2: AChE activity evaluation in presence of ATCh (500 µM) and ascending volumes of rivastigmine for the determination of the IC50, Figure S3: The effect of different concentrations of insulin vs. AChE activity, Figure S4: Predicted versus actual data for the quadratic model (R^2^ ≤ 0.2).
