# Supplementary material for: The Potential Effect of Insulin on AChE and Its Interactions with Rivastigmine In Vitro
Source: Pharmaceuticals (Basel). 2021 Nov 9;14(11):1136. doi: 10.3390/ph14111136 (PMC8617642; doi:10.3390/ph14111136)
Supplement: Supplementary file 1 [file pharmaceuticals-14-01136-s001.zip › pharmaceuticals-1432656-supplementary.pdf]

Supplementary Materials: The following are available online at www.mdpi.com/xxx/s1, Figure S1: title, Table S1: title, Video S1: title.

Table S1. Quadratic model as the best model defining the factor responses**.**

**Source**	**Sequential p-value**	**Lack of Fit p-value**	**Adjusted R^2**	**Predicted R^2**	
Linear	0.0002	0.0005	0.6334	0.1914	
2FI	< 0.0001	0.0988	0.9637	0.9000	
Quadratic	0.0505	0.2284	0.9776	0.9453	Suggested
Cubic	0.1558	0.4307	0.9859	-5.0909	Aliased

Table S2. Coded- form:.

**Activity**	=
+0.1658	
-0.0255	A
+0.1973	B
-0.1834	C
-0.0276	AB
+0.0025	AC
-0.1789	BC
+0.0422	C^2

The coefficients of the sentences in the coded equation are used to predict the effect of each variable on the response. Thus, any sentence that has a larger coefficient in the coded equation has a more severe effect on the response.

Table S3. Non-coded form (Actual):**.**

**Activity**	=
+0.015634	
-0.000016	[Insulin.]
+0.000807	[ATCh.]
-0.000023	[Rivastigmine.]
-2.20600E-06	[Insulin.] * [ATCh.]
+2.50000E-08	[Insulin.] * [Rivastigmine.]
-8.94375E-08	[ATCh.] * [Rivastigmine.]
+2.63878E-09	$[\text{Rivastigmine.}]^2$

The non-coded equation is also used to calculate the response in other values of variables (within the design area). With a non-coded equation, the response value in the points where have not been measured can be calculated. At last, analysis of variance (ANOVA) of each response was evaluated and the effects of independent variables were expressed in 3D response plots.

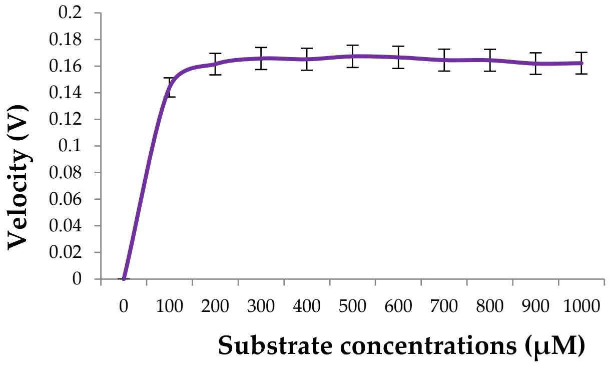

Figure S1. Km determination for ATCh, increasing concentrations of ATCh caused higher AChE activity up to 100 μ above which there was no change. (Km=38.34, Vmax= 5.724e-005, R2=0.9800). All the tests were conducted based on Ellman's method three times, otherwise stated.

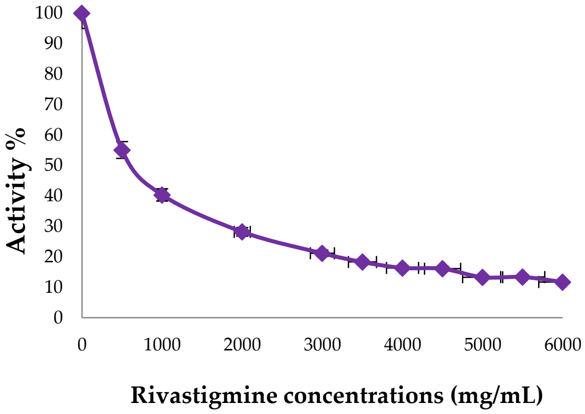

Figure S2. AChE activity evaluation in presence of ATCh (500 μM) and ascending volumes rivastigmine for determination the IC50. The increasing concentrations of rivastigmine caused decreasing AChE activity up to 1200 μM concentration above which there was no more inhibition (Rivastigmine IC50=595, R2= 0.9986).

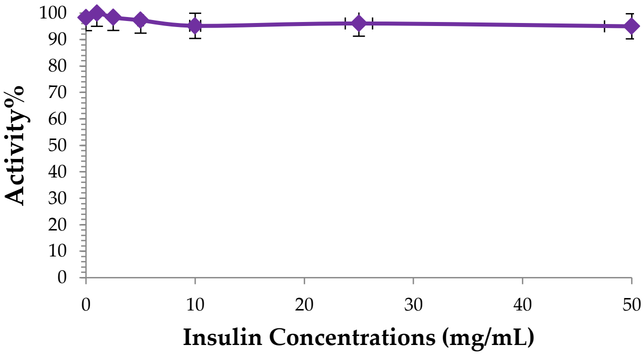

Figure S3. The effect of different concentrations of insulin vs. AChE activity. The 0-50 μM concentrations of insulin were added to the enzyme reaction in 500 μM ATCh concentration. No significant effect was observed even at high concentrations of insulin (σ2=0.00001). The variance of the AChE activity in ascending concentrations of insulin is 0.00001, which means that the dispersion of data is negligible and hence the effect of insulin is insignificant.

Data (AChE activities in ascending concentrations of insulin): 0.3091, 0.3287, 0.3243, 0.3378, 0.3255, 0.3072, 0.3240

The average of the data: $\bar{\boldsymbol{x}} = \frac{\sum_1^7 x_i}{\mathbf{7}} = \mathbf{0 \cdot 3223}$

The variance of the data: $\sigma^2 = \frac{\sum_1^7 x_i - \bar{x}}{\mathbf{7}}$ = 0.00001
